# Disease-specific crosstalk of *Alistipes* with lipoprotein profiles in overweight individuals at high cardiometabolic risk

**DOI:** 10.1038/s41598-026-36024-0

**Published:** 2026-02-13

**Authors:** Amanda Cuevas-Sierra, Andrea Higuera-Gómez, Begoña de Cuevillas, Núria Amigó, María Martínez-Urbistondo, Raquel Castejón, J. Antonio Vargas, J. Alfredo Martínez

**Affiliations:** 1 Precision Nutrition and Cardiometabolic Health, IMDEA-Nutrition Institute (Madrid Institute for Advanced Studies), Campus of International Excellence (CEI) UAM+CSIC, 28049 Madrid, Spain; 2 Department of Pharmacy and Nutrition, Faculty of Biomedical and Health Sciences, Universidad Europea de Madrid-Campus de Villaviciosa de Odón, UAM+CSIC, 28049 Madrid, Spain; 3 Biosfer Teslab, IISPV, CIBERDEM, University Rovira i Virgili. Plaça de Prim, 10, 2° 5ª, 43201 Reus, Tarragona, Spain; 4 Internal Medicine Service of Puerta de Hierro, Majadahonda University Hospital, 2822 Madrid, Spain; 5 Centro de Medicina y Endocrinología, Universidad de Valladolid, Valladolid, Spain; 6 Centro de Investigación Biomédica en Red de La Fisiopatología de La Obesidad y Nutrición (CIBERobn), Instituto de Salud Carlos III, Madrid, Spain

**Keywords:** Alistipes- interactions, Small dense LDL, Advanced lipoprotein profiling, Cardiovascular risk, Metabolic syndrome, Systemic lupus erythematosus, Biomarkers, Diseases, Medical research, Risk factors

## Abstract

Metabolic syndrome (MS) and systemic lupus erythematosus (SLE) represent two pathophysiologically distinct chronic conditions associated with elevated cardiovascular risk. Emerging evidence links gut microbiota to host lipid metabolism and lipoprotein function. This investigation aimed to analyze disease-specific relations between gut microbiota and an advanced lipoprotein profiling in an overweight population affected by MS and SLE. A total of 85 individuals with MS and 66 with SLE, all overweight, were included. Anthropometric, body composition, and biochemical parameters were assessed. Lipoprotein profiles were quantified by^1^H-NMR spectroscopy, and gut microbiota composition via 16S rRNA sequencing. Machine learning Boruta feature selection identified microbial taxa linked to lipid traits. Regression models evaluated microbiota–LDL particle associations by disease type. Participants in the MS group showed more unfavorable values in anthropometric, body composition and clinical biochemistry. Advanced lipoprotein analysis revealed that MS individuals had higher levels of IDL-C, VLDL-TG, small LDL-P, and total LDL-P, with decreased HDL-C, reflecting a more atherogenic profile. Gut microbiota analysis identified *Alistipes* as a key discriminant taxon. A significant association was observed between *Alistipes* abundance and small LDL particle concentrations, being modulated by disease type, suggesting that the association between gut microbiota and lipid metabolism may differ by disease type. Participants with metabolic syndrome showed unfavorable anthropometric, clinical, and lipoprotein profiles, along with distinct gut microbiota, compared to individuals with the autoimmune-driven SLE. The results highlight condition-specific host–microbiota–lipid relationship and supports the use of novel approaches to guide precision strategies for cardiovascular risk reduction in high-risk populations.

## Introduction

Systemic Lupus Erythematosus (SLE) and Metabolic Syndrome (MS) represent distinct clinical conditions with complex etiologies, yet both are characterized by chronic inflammation and features that have been linked to disruptions in lipid metabolism and gut microbiota composition^1,2^. SLE, an autoimmune disease, is marked by systemic inflammation and immune dysregulation, often involving abnormal production of autoantibodies and inflammatory cytokines that impact multiple organ systems^3^. Conversely, MS is a metabolic disorder defined by a cluster of conditions—obesity, increased waist circumference, dyslipidemia, hypertension, and insulin resistance—that elevate the risk of cardiovascular disease and type 2 diabetes^4,5^. Hypertriglyceridemia and low HDL-c are frequently associated with small dense low-density lipoprotein (LDL-c); accordingly, a preponderance of small dense LDL-c has been described in MS^5^. LDL particles constitute a heterogeneous mixture of lipoproteins differing in density, size, lipid composition, electrical charge, and pathological properties^6^. Determination of lipoprotein profile including LDL particle size is interesting, because the small-dense LDL particles can be highly atherogenic due to its ability to penetrate the arterial wall, a low affinity for the receptor, thereby increasing its half-life plasma and low resistance to oxidative stress^7,8^ and presence of Triglyceride rich remnant particles (small VLDL and IDL particles) that contribute on cholesterol deposition in arterial walls beyond LDL-c levels^9^. Traditional diagnostic and therapeutic approaches that focus solely on LDL-c often overlook this LDL subfraction, potentially underestimating cardiovascular risk. Additionally, recent research has highlighted the integral role of gut microbiota in modulating both immune responses and metabolic processes, thereby connecting the gastrointestinal tract to systemic inflammation and metabolic health^10,11^. The gut microbiota contributes to lipid metabolism through the production of bioactive molecules, such as short-chain fatty acids (SCFAs), secondary bile acids, and various lipid derivatives, which can influence lipid profiles and inflammatory responses in distant tissues^12^. Dysbiosis, an imbalance in gut microbial communities, is increasingly recognized in both SLE and MS, where distinct shifts in microbial taxa have been observed^13–15^. This dysbiosis may drive disease pathology by disrupting metabolic homeostasis and exacerbating systemic inflammation, particularly through lipid metabolism pathways, suggesting a "gut-serum lipid axis"^16^that links gut microbiota composition to serum lipid profiles in ways that are specific to each disease state^16,17^. In SLE, microbial imbalances have been linked to immune dysregulation and increased systemic inflammation^18^. These microbial alterations can influence the host lipidome, as certain gut bacteria produce metabolites that impact immune cell function and cytokine production, potentially exacerbating inflammation in these types of patients^18,19^. On the other hand, in MS, dysbiosis often features reductions in SCFA-producing bacteria and increases in lipopolysaccharide-producing species, which can disrupt gut barrier integrity and promote metabolic endotoxemia, thereby fueling systemic inflammation and metabolic dysfunction^20,21^. These findings suggest that while both SLE and MS involve gut microbiota alterations, the specific bacterial taxa and their metabolic outputs may distinctly influence inflammatory processes across these conditions^22^. Among gut microbial genera, *Alistipes* has received growing attention for the associations with lipid metabolism and immune regulation. For example, Zeng et al. (2025) reported that *Alistipes shahii* was inversely correlated with elevated triglyceride levels in human cohorts with dyslipidemia^23^. Moreover, *Alistipes timonensis* has been associated with markers of inflammation in humans, reinforcing the potential role at the immunometabolic interface^24^. In the context of SLE, alterations in *Alistipes* abundance have also been reported compared with healthy controls^25^. By contrast, other genera such as *Bifidobacterium* are well established as beneficial modulators of lipid metabolism, with evidence showing inverse associations with BMI, triglyceride levels, and hepatic fat deposition in both human and animal studies^26^.

Interestingly, recent technological advancements have facilitated swift, dependable, and detailed assessments of circulating lipoproteins. Using proton nuclear magnetic resonance (^1^ H NMR) offers the unique benefit of concurrently assessing lipoprotein particles’ quantity, dimensions, and constitution, thereby affording a more comprehensive insight into alterations linked to metabolic dysregulations^27,28^. In this context, this study seeks to map the inflammatory landscapes of SLE and MS by examining variations in gut microbiota composition and serum lipid profiles in each disease state.

Thus, this study aimed to investigate disease-specific interactions between gut microbiota composition and advanced serum lipoprotein profiles in MS and SLE—two chronic inflammatory conditions with distinct pathophysiological mechanisms but elevated cardiovascular risk. By integrating^1^H-NMR lipid profiling with gut microbiota composition analysis, we seek to identify unique microbiota–lipid interactions that contribute to disease-specific inflammatory and cardiometabolic phenotypes. Ultimately, this work aimed to uncover new insights through a multi-omics approach that could inform the development of tailored biomarkers and targeted interventions for cardiovascular risk management in these chronic conditions.

## Results

### MS patients present a more adverse metabolic and cardiometabolic risk profile, whereas SLE patients exhibit stronger inflammatory activity

This investigation included a total of 151 participants who met with the inclusion criteria for this analysis, predominantly women (70.8%) with a mean age of 57.2 (10.1). The baseline demographic and body composition variables between the SLE and MS groups revealed several significant differences, shown in Table 1.

**Table 1:** Baseline demographic, body composition and anthropometric variables between SLE and MI groups from METAINFLAMMATION cohort.

	Metabolic syndrome (MS) (n = 85)	Lupus erythematosus systemic (SLE) (n = 66)	P value
Age (y)	60.2 (10.3)	53.4 (10.7)	< 0.001
Female sex (%)	45 (52.9)	62 (93.9)	< 0.001
Weight (kg)	88.7 (16.2)	73.9 (16.9)	< 0.001
BMI (kg/m^2^)	31.7 (4.1)	28.4 (5.7)	< 0.001
Waist (cm)	110.2 (10.5)	97.6 (14.0)	< 0.001
Total muscle mass (kg)	52.8 (10.6)	43.8 (6.6)	< 0.001
Total fat (%)	37.3 (6.9)	36.2 (9.0)	0.41
Visceral fat	14.3 (4.7)	8.9 (4.1)	< 0.001
SBP (mmHg)	139 (18)	128 (18)	< 0.001
DBP (mmHg)	80 (12)	75 (11)	0.01
Adherence to Mediterranean diet	6.9 (0.3)	7.1 (0.2)	0.33
Prevalence of obesity (n,%)	60 (70.5)	25 (37.8)	0.03
Prevalence of type 2 diabetes (n,%)	15 (17.6)	3 (4.5)	0.01
Prevalence of hypertension (n,%)	45 (52.9)	13 (19.7)	< 0.001
Prevalence of dyslipidemia (n,%)	36 (42.3)	15 (22.7)	0.01

Data presented as mean (x¯), standard deviation (SD), and p values. The significance threshold was set at p < 0.05, t-test was used to compare the mean of continuous variables and Chi-square (χ 2 ) to compare categorical variables. BMI, Body Mass Index; DBP, diastolic blood pressure; MS, metabolic syndrome; SBP, systolic blood pressure; SLE, systemic lupus erythematosus.

The MS group was significantly older and showed a higher prevalence of obesity, diabetes mellitus, hypertension, and dyslipidemia (p < 0.05), aligning with the metabolic and cardiovascular risk factors characteristic of this condition. Anthropometrically, individuals with MS showed significantly higher BMI, body weight, visceral fat and waist circumference (p < 0.001), alongside increased muscle mass (p < 0.001). Blood pressure was also elevated in the MS group, with both systolic and diastolic measures significantly higher (p < 0.01). Both groups of comparison presented overweight (IMC > 25.0 kg/m^2^).

In the comparison of biochemical, inflammatory, and immunological variables between the MS and SLE groups, several statistically significant differences were found (Table 2). Participants in MS group showed significantly worst glycemic parameters compared to SLE group. Specifically, fasting glucose levels were lower (*p* < 0.001), as were glycated hemoglobin values (*p* = 0.001). Although insulin levels showed a trend toward reduction, the difference was not statistically significant (*p* = 0.06). No significant results were found for HOMA-IR. Lipid profile analysis revealed a significant increase in HDL-cholesterol (*p* = 0.02) and a marked decrease in triglycerides (*p* = 0.006), while total cholesterol remained comparable between groups (*p* = 0.43). Liver function biomarkers showed significant reductions in GPT (*p* < 0.001) and GGT (*p* = 0.002), alongside decreased bilirubin levels (*p* = 0.002). GOT levels did not differ significantly (*p* = 0.99). Additional biochemical data showed significantly higher uric acid levels in MS group (*p* < 0.001). In terms of inflammatory markers, interleukin-6 (IL-6) levels were significantly higher in the SLE group (*p* = 0.04), indicating a more pronounced inflammatory status. Ferritin levels were significantly lower in SLE compared to MS (*p* = 0.002), suggesting differences in iron storage or inflammatory response profiles. Regarding immunological and hematological variables, the MS group showed a higher percentage of leukocytes (p = 0.02), and neutrophils (p = 0.04) compared to SLE. Hematocrit was significantly higher in the MS group (p < 0.001), while the mean corpuscular volume (MCV) was lower (p < 0.001). ESR was higher in the SLE group than in MS, demonstrating differences in hematological markers across the two conditions.

**Table 2:** Blood biochemical, inflammatory and immunological variables between SLE and MI groups from METAINFLAMMATION cohort.

	*Hospital reference values*	Metabolic syndrome (MS) (n = 85)	Lupus erythematosus systemic (SLE) (n = 66)	P value
	***General biochemistry***			
Glucose (mg/dL)	60–100	102 (15)	91 (13)	< 0.001
Glycated hemoglobin (%)	4.5–6.4	5.7 (0.6)	5.4 (0.4)	0.001
Insulin (µU/mL)	2–29.1	11.9 (0.9)	9.5 (7.1)	0.06
HOMA-IR	< 2.5	2.9 (0.2)	2.3 (0.2)	0.15
Total cholesterol (mg/dL)	150.0–200.0	185 (33)	180 (33)	0.43
HDL-cholesterol (mg/dL)	50.0–90.0	52 (16)	58 (15)	0.02
Triglycerides (mg/dL)	30.0–200.0	128 (48)	103 (59)	0.006
GOT (U/L)	6.0–40.0	23.7 (6.2)	23.7 (9.1)	0.99
GPT (U/L)	6.0–40.0	29.6 (10.7)	22.3 (12.2)	< 0.001
GGT (U/L)		33.5 (27.4)	21.4 (15.5)	0.002
Uric acid (mg/dL)	2.5–6.0	5.7 (1.4)	4.6 (1.2)	< 0.001
	***Inflammatory variables***			
LDH (U/L)	120.0–246.0	185.7 (32.2)	188.2 (36.9)	0.65
PCR (mg/L)	0.1–10.0	3.7 (3.9)	5.5 (8.8)	0.11
IL-6 (pg/mL)	0.0–4.4	2.2 (2.1)	3.2 (3.6)	0.04
Ferritin (ng/mL)	5.0–204.0	151.2 (116.2)	86.3 (74.7)	0.002
Fibrinogen (mg/dL)	200.0–400.0	365.3 (86.1)	370.9 (124.6)	0.62
D-dimer (ng/mL)	0.0–500.0	358.9 (197.3)	353.0 (199.4)	0.68
Neutrophils/lymphocyte ratio	0.78 – 3.5	1.8 (0.7)	2.5 (0.9)	0.03
	***Hematological variables***			
Leukocytes (× 10^3^/µL)	4.0–11.5	6.4 (1.6)	5.8 (2.4)	0.02
Neutrophils (%)	1.5–7.5	3.9 (1.1)	3.6 (2.0)	0.04
Lymphocytes (%)	1.2–4.0	2.1 (0.6)	1.4 (0.5)	0.37
Platelets (× 10^3^/µL)	150.0–400.0	234.3 (48.4)	230.8 (61.6)	0.06
Hematocrit (%)	37.0–47.0	45.5 (3.7)	42.7 (2.9)	< 0.001
Mean Corpuscular Volume (fL)		90.7 (7.5)	91.8 (4.1)	< 0.001
ESR (mm/h)		9.8 (8.1)	16.5 (16.2)	0.04

Data presented as mean (x¯), standard deviation (SD), and p values. The significance threshold was set at p < 0.05, t-test was used to compare the mean of continuous variables and Chi-square (χ 2 ) to compare categorical variables. ESR, erythrocyte sedimentation rate; IL-6, interleukin-6; LDH, lactate dehydrogenase; MS, metabolic syndrome; SLE, systemic lupus erythematosus.

### MS exhibits a more atherogenic lipoprotein profile with increased remnant particles and elevated small dense LDL compared to SLE

The analysis of lipoprotein subfractions revealed that individuals with MS exhibited a markedly more atherogenic lipid profile compared to patients with SLE (Table 3). Patients in the MS group displayed significantly higher concentrations of very-low-density and intermediate-density lipoproteins (remnant lipoprotein particles), both cholesterol- and triglyceride-rich fractions. Notably, levels of very-low-density lipoprotein triglycerides (VLDL-TG, p = 0.001), intermediate-density lipoprotein triglycerides (IDL-TG, p = 0.002), and total very-low-density lipoprotein particles (VLDL-P, p = 0.001) were markedly elevated in the MS group. Furthermore, small VLDL particles (small VLDL-P)—a subclass with high atherogenic potential—were significantly increased (p < 0.001), along with large VLDL-P (p = 0.003). A similar trend was observed in LDL subfractions. Small LDL-P, which are independently associated with accelerated atherosclerosis, were significantly higher in the MS group (p < 0.001), contributing to an overall increase in total LDL particle number (p = 0.01). Compared to SLE, MS patients had lower concentrations of medium HDL particles (p < 0.001) and higher levels of small HDL particles (p = 0.001), which are less efficient in cholesterol efflux and exhibit reduced anti-inflammatory capacity. In addition, particle size analysis showed a significant decrease in the mean diameter of LDL and HDL particles in MS (p < 0.001; HDL-Z: p < 0.001), indicative of more atherogenic lipoprotein remodeling (Table 3). Furthermore, patients with SLE exhibited significantly larger average particle diameters across all lipoprotein classes—VLDL-Z (p < 0.001), LDL-Z (21.2 ± 0.2 vs. 21.0 ± 0.3 nm, p < 0.001), and HDL-Z (p < 0.001).

**Table 3:** Comparison of the lipoprotein profile assessed by HNMR-1 between SLE and MI groups from METAINFLAMMATION cohort.

*Outcomes*	Metabolic syndrome (MI) (n = 85)	Lupus erythematosus systemic (SLE) (n = 66)	*P* value
VLDL-C (mg/dL)	16.4 (8.7)	13.8 (8.9)	0.07
IDL-C (mg/dL)	11.4 (3.7)	9.7 (3.9)	0.007
LDL C (mg/dL)	121.9 (27.9)	115.1 (25.9)	0.12
HDL-C (mg/dL)	52.1 (16.3)	58.1 (11.2)	0.02
VLDL-TG (mg/dL)	72.4 (30.3)	56.5 (28.1)	0.001
IDL-TG (mg/dL)	12.2 (3.0)	10.6 (3.3)	0.002
LDL-TG (mg/dL)	16.1 (4.2)	15.8 (4.7)	0.78
HDL-TG (mg/dL)	16.0 (4.7)	15.6 (5.8)	0.65
VLDL-P (nmol/L)	52.6 (22.9)	41.1 (20.7)	0.001
Large VLDL-P (nmol/L)	1.4 (0.5)	1.1 (0.4)	0.003
Medium VLDL-P (nmol/L)	5.1 (2.3)	4.7 (2.8)	0.42
Small VLDL-P (nmol/L)	46.1 (20.4)	35.2 (17.5)	< 0.001
LDL-P (nmol/L)	1236.9 (260.4)	1135.4 (232.1)	0.01
Large LDL-P (nmol/L)	185.3 (39.9)	192.8 (36.5)	0.23
Medium LDL-P (nmol/L)	377.3 (123.3)	361.9 (119.1)	0.44
Small LDL-P (nmol/L)	674.3 (126.1)	580.6 (98.4)	< 0.001
HDL-P (μmol/L)	28.2 (4.2)	27.6 (5.3)	0.49
Large HDL-P (μmol/L)	0.28 (0.03)	0.29 (0.04)	0.20
Médium HDL-P (μmol/L)	9.4 (1.6)	10.8 (2.3)	< 0.001
Small HDL-P (μmol/L)	18.5 (3.5)	16.5 (4.1)	0.001

TG: triglycerides; C: cholesterol; HDL: high density lipoprotein; IDL: intermediate density lipoprotein; LDL: low density lipoprotein; VLDL: very low density lipoprotein; P: particles.

Statistically significant differences in lipoprotein particle sizes were observed between patients with MS and those with SLE. VLDL particle size (VLDL-Z) was slightly larger in SLE patients (42.2 ± 0.1 nm) compared to those with MS (42.1 ± 0.2 nm; *p* < 0.001). Likewise, LDL particle size (LDL-Z) was 21.0 ± 0.3 nm in the MS group and 21.2 ± 0.2 nm in the SLE group (*p* < 0.001), and HDL particle size (HDL-Z) was 8.2 ± 0.06 nm and 8.3 ± 0.09 nm (*p* < 0.001), respectively (Table 3).

**Fig. 1:**
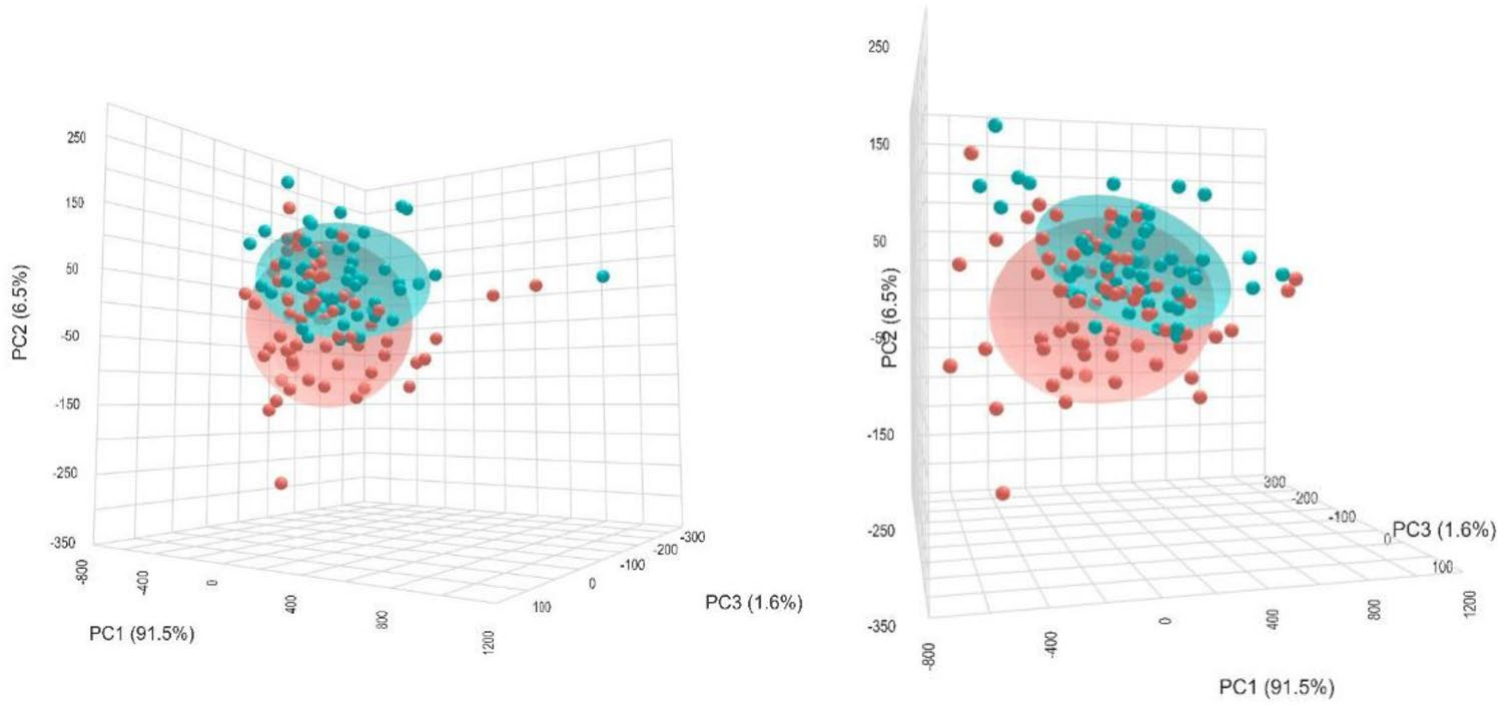
Principal component analysis (PCA) of lipoprotein profiles in individuals with metabolic syndrome (MS) and systemic lupus erythematosus (SLE). Three-dimensional PCA score plot illustrating the distribution of individual subjects based on their plasma lipid profile. Each point represents a single participant, with red and blue spheres corresponding to MS and SLE groups, respectively. The first three principal components (PC1, PC2, and PC3) explain 91.5%, 6.5%, and 1.6% of the total variance, respectively. Ellipsoids represent 95% confidence intervals for each group. The partial separation along PC1 indicates distinct lipoprotein signatures between groups, with greater dispersion observed in the MS group, suggestive of higher intra-group heterogeneity in lipid metabolism.

The three-dimensional PCA plot (Fig. 1) shows the distribution of individual samples based on the first three principal components: PC1 (91.5%), PC2 (6.5%), and PC3 (1.6%), cumulatively explaining 99.6% of the total variance in the dataset. Samples clustered primarily along PC1, indicating that this component captures the dominant variance associated with lipid profile differences. Although there is partial overlap between the two ellipsoids, the separation along PC1 suggests a distinguishable lipoprotein signature in individuals with metabolic syndrome compared to those with SLE. The tighter clustering of the SLE group and broader spread of the MS group indicate higher heterogeneity in lipid composition within the MS cohort.

### SLE patients show reduced gut microbial alpha diversity and a distinct community composition versus MS

**Fig. 2:**
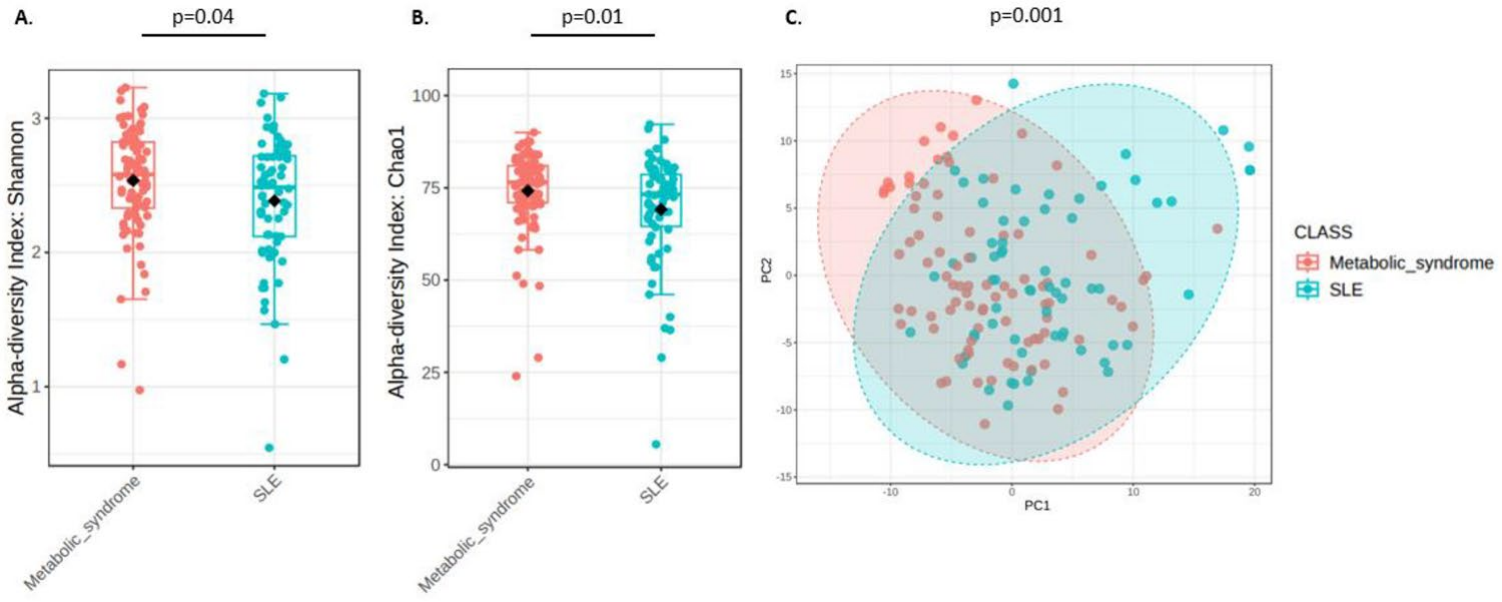
Comparison of gut microbiota diversity between participants with Metabolic Syndrome (MS) and Systemic Lupus Erythematosus (SLE). Alpha diversity is represented as box plots, with each point indicating an individual participant’s value. **A**. Alpha diversity indices (Shannon) for both groups, indicating that MS patients exhibit significantly higher alpha diversity (p = 0.04). **B**. Comparison of Chao1 index between both groups (*p* = 0.01). **C**. Principal Component Analysis (PCA) based on beta diversity, highlighting the distinct microbial community structure between the two conditions (*p* = 0.001, PERMANOVA test). Each point represents an individual sample, and the colored ellipses denote the 95% confidence interval for each group. SLE and Metabolic Syndrome samples cluster separately, indicating significant compositional differences in their gut microbiota.

Microbial diversity was evaluated by the Shannon index and Chao1, showing a a significant decrease in SLE participants (Fig. 2A and 2B). Beta diversity analysis was performed by the Bray–Curtis method. The PCA plot showed clustering according to the groups and significantly different microbial composition between MS and SLE (PERMANOVA p value = 0.011) (Fig. 2C).

**Fig. 3:**
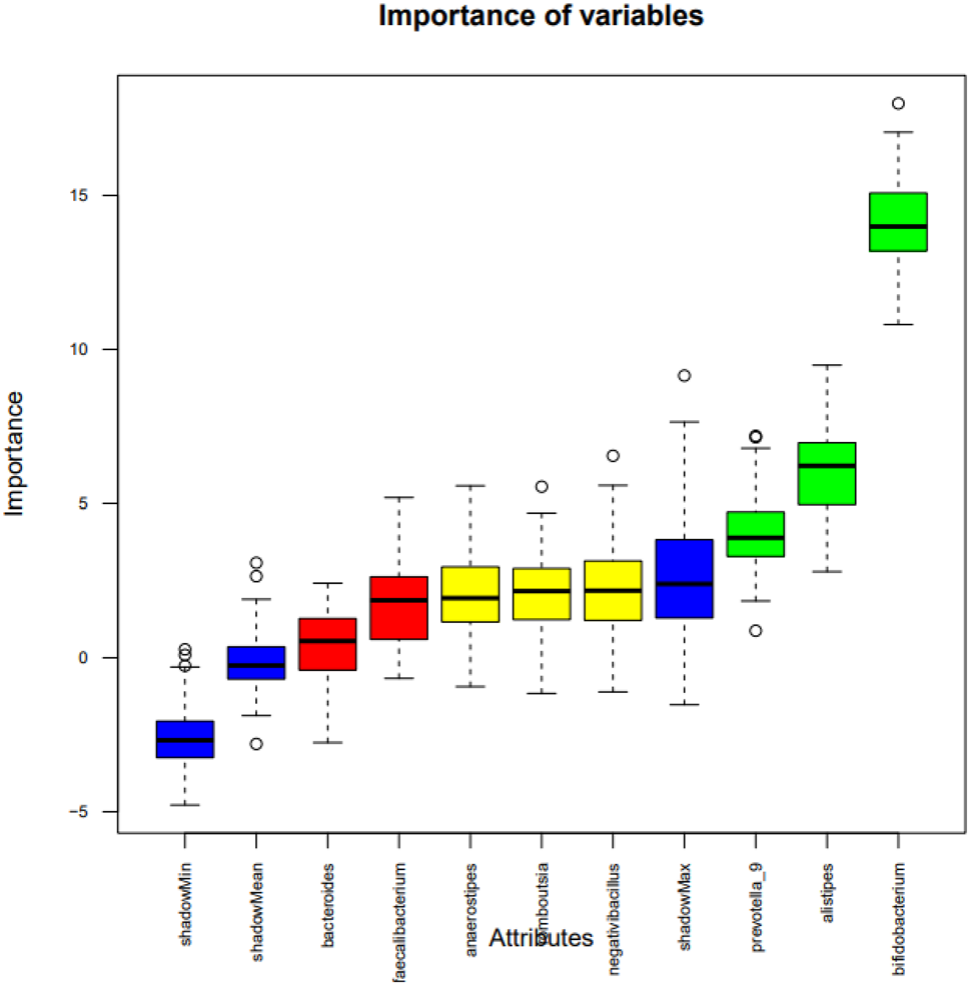
Variable importance ranked by the Boruta algorithm. The Boruta algorithm identifies relevant features by comparing their importance to that of randomized shadow variables, with higher scores indicating greater contribution to the classification model. The boxplots display the distribution of importance scores for each variable across multiple iterations of the Boruta feature selection process. Variables on the right show higher importance, indicating a stronger contribution to the model’s predictive performance. Shadow variables (in blue) serve as references for identifying truly relevant attributes. Variables with importance scores significantly higher than shadow variables are considered important.

In addition, Fig. 3 presents a box graph illustrating the importance of the variables used in the analysis. The variable Bacteroides displayed low importance scores, comparable to the randomized shadow features in the Boruta analysis, indicating that it contributed minimally to the predictive model relative to other genera such as *Alistipes* and *Bifidobacterium.* Variables represented by green-colored boxes exhibited the highest importance scores, highlighting attributes such as Alistipes and Bifidobacterium. This representation distinguishes the most relevant variables from those with lower contributions, providing a illustrative overview of factors driving the predictive model.

**Fig. 4:**
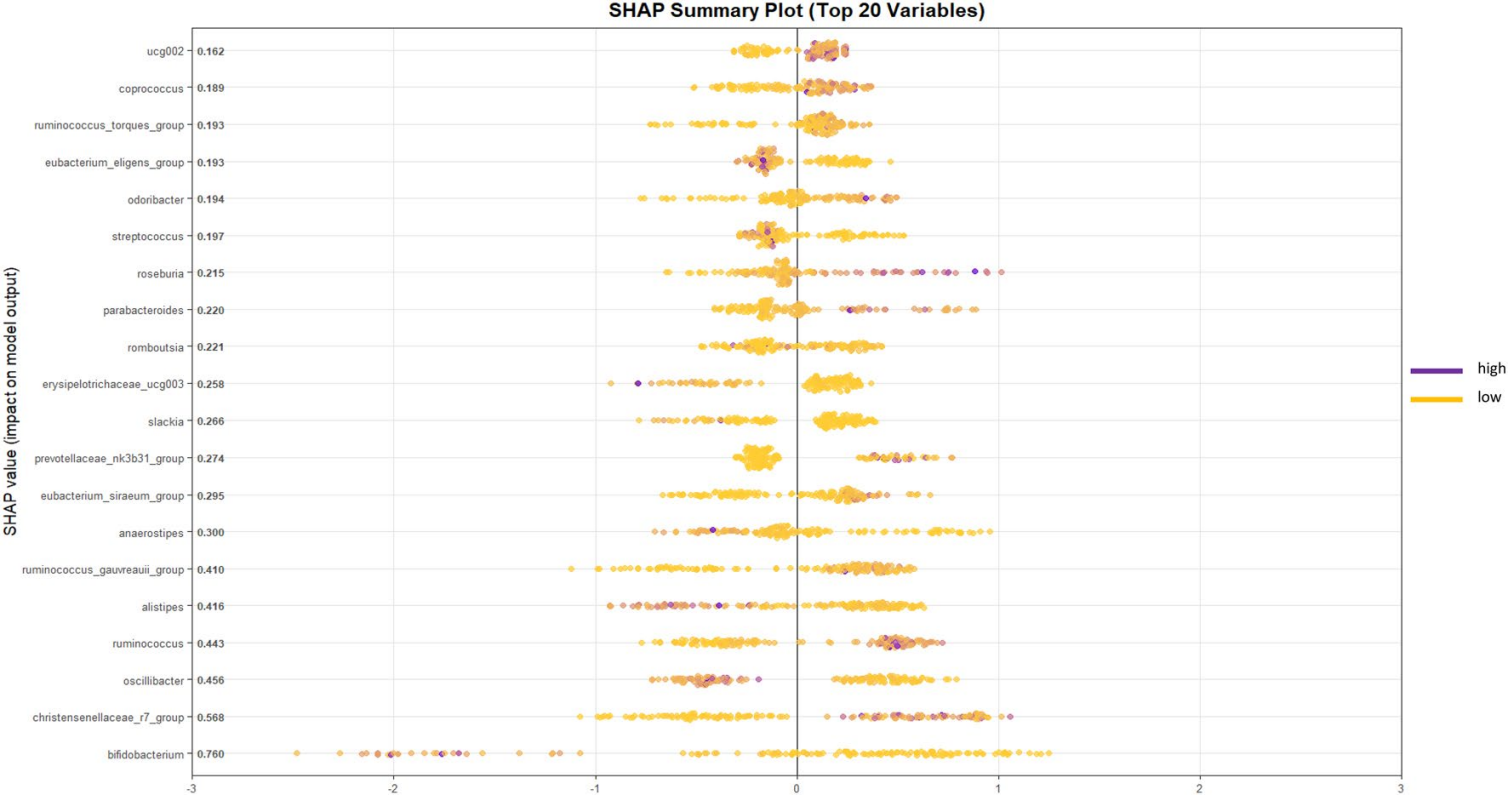
Summary SHAP plot of the 20 most influential bacterial genera in the XGBoost model. Each point represents one sample, with color indicating the normalized abundance of each genus (yellow = low, purple = high). The x-axis shows the SHAP value, reflecting the direction and magnitude of each feature’s impact on the model output. Positive SHAP values indicate higher associated predicted probability of metabolic syndrome, while negative values indicate lupus. The genus Alistipes (mean |SHAP|= 0.416) showed lower abundances associated with the metabolic syndrome group.

Figure 4 displays the 20 taxa with the highest mean absolute SHAP values, representing the strongest contributors to the model’s predictions. Among these, the genus *Alistipes* showed an intermediate contribution (mean |SHAP|= 0.314). In the plot, positive SHAP values correspond to a higher predicted probability of MS, whereas negative SHAP values indicate a stronger prediction for SLE. Low relative abundances of *Alistipes* (yellow points) were mainly associated with positive SHAP values, suggesting that reduced levels of this genus are linked to the MS phenotype. Conversely, higher abundances (purple points) tended to shift SHAP values toward the negative axis, indicating a potential protective or modulatory role of *Alistipes* in the SLE group.

## Alistipes abundance shows disease-specific interaction with small LDL particle concentrations

To further explore the potential interplay between host lipid metabolism and gut microbiota in chronic inflammatory diseases, we performed an integrative analysis combining lipoprotein subfraction data and gut microbial profiles in patients with MS and SLE. Given the distinct lipoprotein and microbial signatures observed in each group, we aimed to determine whether disease-specific patterns of association exist between these two omics layers.

**Fig. 5:**
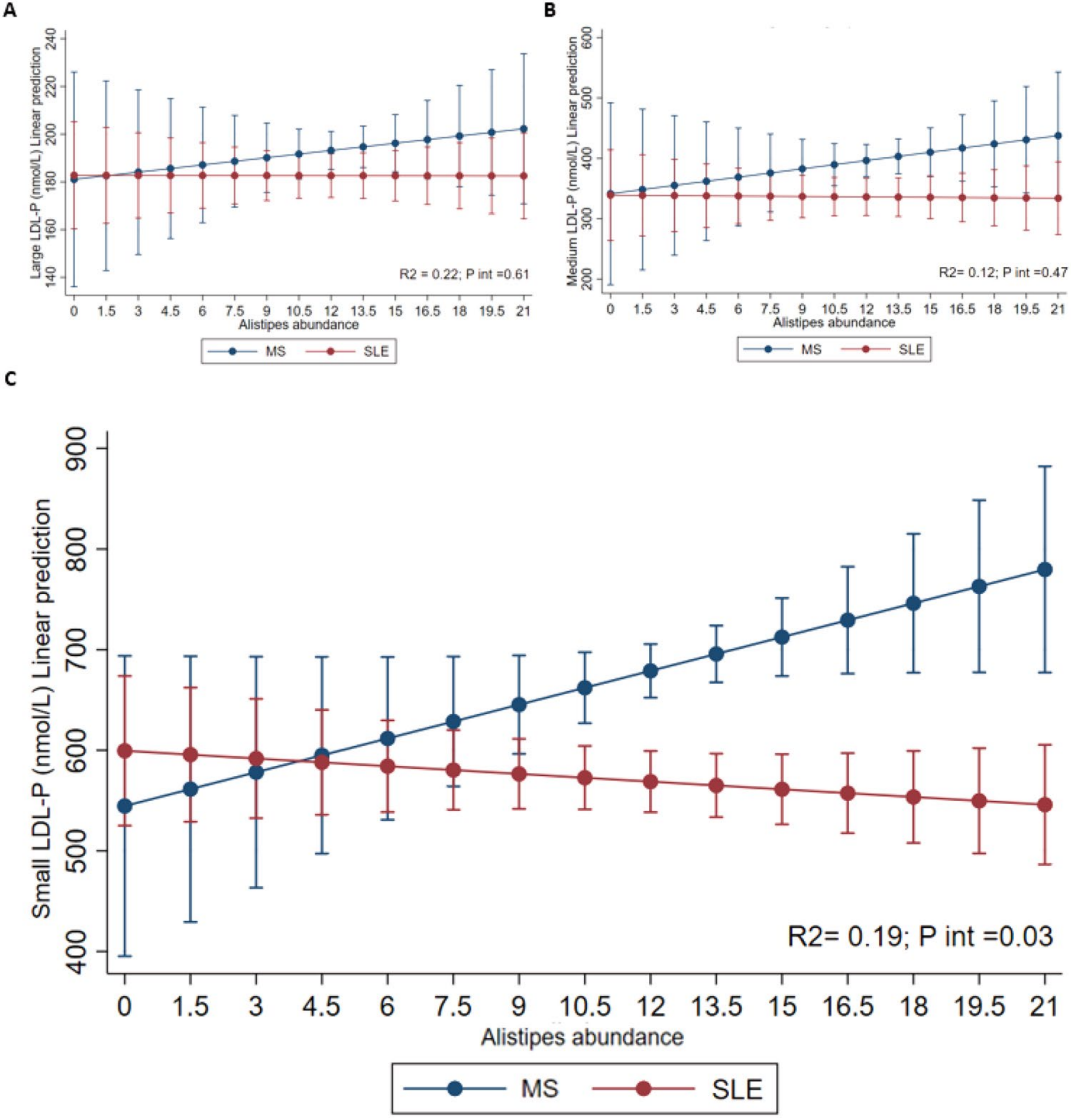
Predicted values of LDL particles (nmol/L) (A, large; B, medium; C, small) in patients with metabolic syndrome (MS) and systemic lupus erythematosus (SLE), according to the abundance of *Alistipes* genus. Blue line represents values for patients with MS and the red line represents values for SLE. The model was adjusted for age, sex, adherence to Mediterranean diet and total cholesterol.

A regression analysis was conducted to evaluate the interaction between Alistipes abundance, type of inflammatory disease (MS vs. SLE), and small LDL particle concentrations. As shown in Fig. 5, higher *Alistipes* abundance was associated with increased predicted small LDL particle concentrations in participants with MS (blue line), whereas in participants with SLE (red line), higher *Alistipes* abundance was associated with a slight decrease in small LDL particle concentrations. This statistically significant interaction (R^2^ = 0.19; p = 0.03) indicates that the relationship between *Alistipes* abundance and LDL particle size differs depending on disease type, reflecting a condition-specific effect modification. In other words, this is an effect modification of predicted values shown in the figure were adjusted for age, sex, total cholesterol, and adherence to the Mediterranean diet, highlighting that the observed interaction is independent of these covariates.

## Discussion

In this investigation of individuals with SLE and MS, we identified distinct clinical, metabolic, bacterial and lipoprotein-related profiles that reflect the differential cardiometabolic risk burden across these two pathophysiological conditions. Despite their shared inflammatory underpinnings, SLE and MS show distinct different cardiometabolic features^29^. Our results also highlight disease-specific lipoprotein and gut microbiota profiles, which are associated with these conditions, although causal relationships cannot be established.

A particularly novel aspect of our study lies in the detailed characterization of lipoprotein subfractions using advanced particle analysis and the integration with gut microbiota results^28,30,31^. Regarding lipoprotein findings, individuals with MS showed a more atherogenic lipoprotein profile, characterized by higher concentrations of IDL-c, VLDL-TG, IDL-TG, VLDL particles, large and small VLDL particles, LDL particles, small LDL particles, and small HDL particles, along with lower levels of HDL-c and medium HDL particles. These patterns are consistent with previous reports linking central fat accumulation and insulin resistance to dyslipidemic features, including elevated plasma triglycerides, increased VLDL and IDL, predominance of small dense LDL particles, and reduced HDL-c^32^. The predominance of small, dense LDL cholesterol (LDL-c) has also been recognized as an emerging cardiovascular risk factor by the National Cholesterol Education Program Adult Treatment Panel III^33^.

Previous studies have found that patients with MS have LDL particle sizes smaller than those without this syndrome^34,35^, even independently of inflammation^36^. LDL levels are associated with increased CVD risk^37,38^, however the LDL particle distribution is more important and should be discussed in the metabolic syndrome^39,40^. The enrichment in small LDL particles—a phenotype associated with increased arterial intima penetration and oxidative susceptibility—likely contributes to the enhanced atherosclerotic cardiovascular disease risk in this group an increased cardiovascular risk^40^. Concurrently, the observed depletion of medium and large HDL particles, along with a shift toward smaller, dysfunctional HDL, suggests impaired reverse cholesterol transport and reduced anti-inflammatory potential.

In contrast, the SLE group demonstrated a more favorable lipoprotein profile, characterized by higher levels of HDL-c and medium HDL particles. This may suggests more favorable lipid profile and atherogenic phenotype, despite the chronic systemic inflammation typically observed in autoimmune conditions like SLE. Interestingly, previous publications have found that HDL-c levels in SLE patients are generally reduced, particularly in those with high disease activity or under corticosteroid treatment. For instance, Zhou et al. (2020)^41^ reported significantly lower HDL-c concentrations in young female SLE patients compared to healthy controls, with HDL-c inversely correlating with disease activity scores (SLEDAI). Leong et al*.* (2000)^42^found that 73% of SLE patients exhibited abnormal lipid profiles, with lower HDL-c particularly associated with high-dose corticosteroid use and renal involvement. More recently, Huang et al*.* (2023)^46^ reported elevated levels of total cholesterol, LDL-c, triglycerides, and ApoB, along with decreased HDL-c and ApoA1 in SLE patients, particularly those with severe inflammation and kidney damage. These discrepancies with our findings may be explained by differences in disease severity, corticosteroid exposure, demographic characteristics, or renal function within the study cohorts. Nevertheless, when compared with the MS group, the lipoprotein profile of SLE patients was markedly less atherogenic despite ongoing inflammation, reinforcing the concept that metabolic syndrome confers a distinct and more pronounced dyslipidemia burden, which may account for the elevated cardiovascular risk typically observed in this condition. Moreover, principal component analysis highlighted a distinct lipoprotein signature between MS and SLE groups. The tighter clustering of the SLE samples suggests a more homogeneous lipid profile, whereas the broader dispersion observed in MS points to considerable inter-individual variability, potentially driven by factors such as obesity, insulin resistance, or genetic predisposition. These findings suggest the need of tailored lipid-lowering strategies and personalized cardiovascular risk management in MS.

Concerning gut microbiota composition, the presence of *Alistipes* was notable. This result was corroborated by the Boruta feature selection algorithm, which ranked *Alistipes* as one of the top contributors to disease classification. *Alistipes* is a genus of gram-negative, anaerobic bacteria within the Bacteroidetes phylum, and has been consistently associated with metabolic and inflammatory disorders^44^. Previous studies have shown that increased abundance of *Alistipes* correlates with obesity, insulin resistance, and elevated pro-inflammatory cytokine profiles^44^. In MS, this presence may reflect gut microbial dysbiosis linked to low-grade systemic inflammation and altered lipid metabolism. Some publications have reported that species of *Alistipes* genus can modulate the host plasma lipidome in mouse model^45^. Similarly, an untargeted lipidomic and 16S rRNA sequencing study in mice by Yasuda et al. (2020)^46^ identified a substantial fraction of gut lipids as microbiota-dependent, including a novel class of α-hydroxylated fatty acids, pointing to direct microbial contributions to host lipidome remodeling. In humans, a study by Castro-Mejía et al. (2022)^47^ demonstrated that specific microbial taxa, such as Lachnospiraceae and Coriobacteriaceae, were positively associated with total cholesterol and large LDL subfractions, while Bacteroidaceae and Bifidobacteriaceae showed negative associations—highlighting the microbiota’s influence on lipoprotein particle size and distribution. Additionally, Liu et al. (2019)^48^ reported altered gut microbiota and lipidomic profiles in women with gestational diabetes and hyperlipidemia, suggesting that microbial shifts may modulate circulating lipid species during metabolic stress. Collectively, these studies show evidence that gut microbial composition is intricately linked with lipidomic patterns, potentially influencing atherogenic lipoprotein and cardiometabolic risk. However, to date, no studies have been found that establish relationships between the abundance of *Alistipes* and the size of LDL particles. Nonetheless, this analysis shows that *Alistipes* abundance is associated with differences in small LDL particle concentrations in a disease-specific manner. This genus was consistently prioritized in the Boruta feature selection, highlighting the relative importance in the predictive model linking gut microbiota and lipoprotein profiles. While the precise mechanisms remain unclear, previous reports suggest a role of *Alistipes* in short-chain fatty acid production, which could plausibly influence lipid metabolism. These observations provide a preliminary view of disease-specific interactions between gut microbiota and lipoprotein subfractions, which require further investigations.

In fact, an intriguing finding of this study was the disease-specific interaction observed between gut microbial composition and lipoprotein subfraction profiles. Specifically, interaction analysis revealed that higher intestinal abundance of *Alistipes* was significantly associated with an increase in small dense LDL particles—but notably, this relationship was restricted to individuals with MS and not observed in patients with SLE. This differential association may reflect an effect modification by disease phenotype, as the relationship between Alistipes abundance and small LDL particles was observed in MS but not in SLE. These results highlight that the interactions between gut microbial communities and lipoprotein profiles may differ depending on the underlying pathophysiological context, suggesting that disease-specific microbiome–lipidome patterns exist that warrant further investigation. In this sense, previous studies have demonstrated that individuals with MS exhibit a higher prevalence of small dense LDL particles, which are considered more atherogenic and are associated with increased cardiovascular risk. Moreover, lifestyle interventions that modify the gut microbiota have been shown to improve lipoprotein profiles and reduce small LDL particle concentrations in MetS patients^49^. These small LDL particles are believed to be more prone to oxidation, leading to endothelial dysfunction and contributing to the pathogenesis of atherosclerosis^40^. Gut microbiota could potentially influence lipoprotein metabolism through several mechanisms, such as fermentation of dietary fiber into short-chain fatty acids (SCFAs), which have been suggested to modulate lipid metabolism in preclinical and human studies^50^. Certain bacterial species, such as *Alistipes*, may promote the synthesis of pro-inflammatory molecules that alter lipid handling and increase the concentration of small, dense LDL particles in specific inflammatory contexts, as seen in MS^51^ Emerging evidence indicates that the gut microbiome can shape the host lipid and lipoprotein metabolism for example, altering the host BA pool^52–55^. In parallel, microbial fermentation of dietary fibers generates short-chain fatty acids (SCFAs; predominantly acetate, propionate and butyrate), which enter portal circulation and serve as substrates and signaling molecules for peripheral tissues^56^. Acetate and butyrate can fuel hepatic de novo lipogenesis and cholesterol synthesis^57,58^, whereas propionate may inhibit these processes^52,59^. SCFAs also modulate adipose tissue: under normal conditions they inhibit lipolysis (reducing free fatty acid flux to the liver)^60^, and SCFA levels are often elevated in obesity-linked dysbiosis^61^. SCFAs and other microbial metabolites further engage enteroendocrine circuits to influence postprandial lipoprotein output^62–64^. Human trials support these findings, showing that dietary or prebiotic interventions that increase beneficial taxa (e.g., *Akkermansia*, *Faecalibacterium*) lower VLDL and LDL fractions and raise HDL in individuals with metabolic syndrome^49^. These pathways provide a framework for understanding how gut microbiota composition may influence host lipoprotein metabolism in MS and SLE, suggesting targets for future mechanistic and therapeutic studies.

Our findings may have some strength and clinical implications. These results revealed that individuals with MS exhibited a more atherogenic lipid profile. In addition, the role of *Alistipes* in lipid metabolism, particularly in relation to LDL particle size, remains underexplored. Our findings contribute to this area by suggesting a potential link between *Alistipes* abundance and the presence of small dense LDL particles in MS patients^65^. Together, these data demonstrate distinct gut microbial profiles in MS and SLE, which may reflect differences in inflammatory status or therapeutic exposures. These microbial patterns could serve as useful markers for further study in relation to disease activity. In addition,^1^H-NMR is a non-destructive technology that requires minimum sample processing to quantify the most abundant metabolites and macromolecules present in different biological matrices, even if these analytes have identical molecular masses. Moreover, this study provides an integrated perspective of gut microbiota together with lipoprotein profiles, offering insights into disease-specific host–microbe associations that could be relevant for cardiometabolic risk. Specifically, a particular strength of this study lies in the identification of an effect modification, suggesting that the influence of the gut microbiome on lipid metabolism is not uniform but rather modulated by the host’s underlying inflammatory and metabolic context. This highlights the importance of disease-specific host–microbiota interactions in shaping lipid profiles and cardiometabolic risk.

This study also presents some limitations. The cross-sectional design of this study precludes the establishment of causal relationships between gut microbiota composition and lipoprotein profiles. Observed associations should therefore be interpreted as correlations, and mechanistic interpretations remain hypothesis-generating. Although we employed robust methodologies for microbiome and lipoprotein profiling, the sample size may limit the generalizability of our findings and reduce statistical power for detecting subtle interactions or rare taxa. Additionally, while 16S rRNA sequencing provides valuable taxonomic information, functional information would be needed to better understand the mechanistic pathways linking microbial taxa with host lipid metabolism. Finally, despite adjusting for key covariates, other lifestyle variables may influence the observed associations.

As conclusion, this investigation revealed distinct differences in anthropometric, body composition, biochemical, lipoprotein, and gut microbiota profiles between two chronic inflammatory conditions—MS and SLE—both characterized by overweight and elevated cardiovascular risk.

MS patients presented a more atherogenic lipoprotein profile, particularly with increased small dense LDL particles, while SLE patients showed a comparatively lower cardiometabolic burden. Cross-sectional analyses revealed disease-specific associations between Alistipes abundance and small LDL particles, which warrant further longitudinal studies to confirm their relevance.

## Methods

### Study design and population

This study is part of the METAINFLAMMATION-CM project (ref. Y2020/BIO-6600), a prospective controlled study designed to investigate the relationship between chronic inflammation and overweight. Participants were consecutively recruited between January 2022 and June 2023 at the Internal Medicine Service of Puerta de Hierro Majadahonda University Hospital (Madrid, Spain). The study protocol was approved by the hospital’s Research Ethics Committee (approval number PI 164–21), conducted in accordance with the Declaration of Helsinki, and all participants provided written informed consent prior to enrollment^66^.

This analysis included a subset of individuals from the METAINFLAMMATION-CM cohort, specifically those for whom both gut microbiota composition and serum metabolomic profiling data were available (n = 151). Participants were categorized into two mutually exclusive groups: those with MS and those with SLE, with no overlap between the two. The MS group included individuals with obesity and metabolic syndrome, diagnosed based on WHO and NCEP ATP III criteria, and characterized by clinical features such as abdominal obesity, insulin resistance, dyslipidemia, and hypertension^67,68^. The SLE group comprised patients fulfilling the EULAR/ACR 2019 classification criteria, who were in a stable clinical condition and under standard immunosuppressive treatment. Disease activity in SLE was assessed using the SLE Disease Activity Index 2000 (SLEDAI-2 K) and the SLICC/ACR damage index. Patients with SLE were classified according to ICD-10 code **M32**, while patients with Metabolic Syndrome (MS) were classified according to ICD-10 code **E88.81**^67,68^.

Inclusion criteria were age > 18 years, BMI between 17.01 and 51.35 kg/m^2^, and a confirmed diagnosis of either MS or SLE by attending physicians. Patients were excluded if they were pregnant or breastfeeding, had severe psychiatric disorders, were taking medications known to significantly affect body weight or microbiota composition, were functionally dependent, or had any condition that might interfere with adherence to study procedures. Anthropometric measurements, body composition and biochemical data were collected in accordance with approved protocols and ethical guidelines^66–68^.

### Anthropometrics measurements

Anthropometric parameters were evaluated by trained dietitians employing standardized and validated procedures^66–70^. Body weight and body composition metrics—including weight, skeletal muscle mass, total fat and visceral adiposity—were obtained using a bioimpedance analyzer (TANITA SC-330; Tanita Corp., Japan). Waist circumference was recorded with a flexible anthropometric tape according to recommended clinical guidelines, ensuring consistency through assessment by trained professionals. Body mass index (BMI) was computed as weight (kg) divided by height in meters squared (m^2^). Blood pressure readings (systolic and diastolic) were taken using a calibrated sphygmomanometer, adhering to internationally recognized protocols. Prevalence of obesity, diabeter type 2, hypertension and dyslipidemia were assessed according to the diagnosis of the physician for each participant. Dietary adherence to the Mediterranean pattern was measured using a validated 14-item questionnaire validated for Spanish population^71–73^.

### Biochemical measurements

Venous blood samples were collected under fasting conditions and analyzed for a range of hematological parameters, including total leukocyte count, lymphocytes, neutrophils, monocytes, mean corpuscular volume, platelets and erythrocyte sedimentation rate (ESR), using an automated hematology system (SYSMEX XN-20; Roche, Basel, Switzerland) in accordance with validated analytical protocols^66–69^. Standard biochemical assays were carried out to determine fasting glucose, total cholesterol, glycated hemoglobin (HbA1c), ferritin, uric acid, gamma-glutamyl transferase (GGT), triglycerides, alanine aminotransferase (ALT), and aspartate aminotransferase (AST). These were processed using a quality-controlled autoanalyzer (Atellica™ Solution, Siemens) and performed according to the hospital’s standard operating procedures. Inflammatory and metabolic biomarkers including C-reactive protein (CRP), fibrinogen, D-dimer, insulin, interleukin-6 (IL-6) were measured primarily via enzyme-linked immunosorbent assay (ELISA) using commercially available kits (Sigma-Aldrich), following the manufacturers’ instructions. Insulin resistance was estimated using the Homeostatic Model Assessment for Insulin Resistance (HOMA-IR), calculated as:

$$\left[ {{\text{Fasting glucose }}\left( {{\mathrm{mg}}/{\mathrm{dL}}} \right) \, \times {\text{ fasting insulin }}\left( {\mu U/{\mathrm{mL}}} \right)} \right] \, / \, 405$$

### ^1^H-NMR analysis

Prior to^1^H-NMR profiling, 200 µL of fasting serum obtained at baseline were mixed with 50 µL of deuterated water and 300 µL of 50 mM phosphate-buffered saline (PBS) adjusted to pH 7.4. Spectral acquisition was performed at 306 K using a Bruker Avance III 600 MHz NMR spectrometer, operating at a proton resonance frequency of 600.20 MHz, as previously described^74^.

### Advanced lipoprotein profiling

Quantification of serum lipoproteins was conducted using the Liposcale® Test, an advanced method based on two-dimensional diffusion-ordered^1^H-NMR spectroscopy (a CE marked technology and clinically used in Spain). This technique deconvolutes the methyl group signal via nine Lorentzian line shapes to determine lipid concentrations across large (L), medium (M), and small (S) subfractions of VLDL, LDL, and HDL. Associated diffusion coefficients were used to estimate subclass-specific particle diameters. Lipid concentrations were integrated with particle volumes to estimate the particle number for each lipoprotein subclass. Finally, the mean particle sizes for VLDL, LDL, and HDL were computed by weighing the diameter of each subclass by its proportion of total particle count. Coefficients of variation ranged from 2 to 4% for particle number and remained below 0.3% for particle diameter^74^.

### Sequencing bacterial DNA for gut microbiota profiling.

Participants self-collected fecal samples using OMNIgene®•GUT kits (DNA Genotek, Ottawa, ON, Canada), following the manufacturer’s instructions. Aliquots were prepared in duplicate (2 mL tubes) and stored at –80 °C until processing. Genomic DNA was extracted using the QIAamp DNA Stool Kit (Qiagen, Hilden, Germany), according to the standard protocol. The V3–V4 hypervariable regions of the bacterial 16S rRNA gene were amplified using specific barcoded primers. PCR reactions (25 μL) were performed with 15 μL of Phusion® High-Fidelity PCR Master Mix (New England Biolabs), 2 μM of each primer, and ~ 10 ng of template DNA. Thermal cycling included initial denaturation at 98 °C for 1 min; 30 cycles of denaturation at 98 °C for 10 s, annealing at 50 °C for 30 s, and extension at 72 °C for 30 s; followed by a final extension at 72 °C for 5 min. PCR products were checked by 2% agarose gel electrophoresis with SYBR Green loading buffer. Amplicons were pooled in equimolar concentrations and purified using the Qiagen Gel Extraction Kit (Qiagen, Germany). Sequencing libraries were prepared with the TruSeq® DNA PCR-Free Sample Preparation Kit (Illumina, USA), and index codes were added according to the manufacturer’s instructions. Library quality was assessed using a Qubit® 2.0 Fluorometer (Thermo Scientific) and Agilent Bioanalyzer 2100 system. The sequencing was performed on an Illumina NovaSeq platform, generating 250 bp paired-end reads. Reads were demultiplexed based on unique barcodes, and primers were trimmed. Paired-end reads were merged using FLASH (v1.2.7)^75^ , and the resulting spliced sequences were referred to as raw tags. Quality filtering was conducted using QIIME (v1.9.1)^76,77^ to obtain high-quality clean tags. Chimeric sequences were identified and removed using the UCHIME algorithm by comparison with reference databases (SILVA for 16S, https://www.arb-silva.de)^36^,^78^ The resulting high-quality tags were denoised using the DADA2 plugin in QIIME2 (v2022.2) to generate amplicon sequence variants (ASVs). Taxonomic assignment was performed with the SILVA database. All the datasets generated and/or analysed during the current study are available in the NCBI Sequence Read Archive (SRA) under accession number PRJNA1279632.

### Statistical analysis

Variables were expressed as means (x) and standard deviations (SD) for quantitative variables and number of cases (n) and proportions (%) for qualitative variables. Normality of the data was assessed by Shapiro–Wilk test. Student’s t and Mann–Whitney tests were implemented depending on normality to compare the means of the continuous variables at the beginning of the study and the categorical variables were statistically screened using the chi-square (χ2 ) test.

For microbiota analysis, alpha diversity was estimated with Shannon and Chao1 indices at the genus level, computed in MicrobiomeAnalyst^79^. Filtering criteria included a minimum count threshold of 4, prevalence ≥ 20% of samples, and exclusion of low-variance features (top 20% based on interquartile range). Group comparisons were performed with the Mann–Whitney U test, and results were visualized as box plots. Beta diversity was evaluated using Bray–Curtis dissimilarity and significance assessed with PERMANOVA, followed by visualization with principal coordinate analysis (PCoA).

To explore lipoprotein variability, principal component analysis (PCA) was performed using the *prcomp* function in R (version 4.1.1; https://www.R-project.org). Lipoprotein features were standardized before analysis. PCA provided an unsupervised overview of the variance structure and facilitated visualization of group-specific lipoprotein signatures. PCA allowed for dimensionality reduction and the visualization of the relationships between lipid profiles in SLE and MS patients. Given that our main objective was to identify taxa predictive of advanced lipoprotein traits across individuals, we employed the Boruta algorithm for feature selection (R package *Boruta*). Boruta is a random-forest wrapper that iteratively compares the importance of original variables to that of permuted “shadow” variables, thereby identifying all relevant predictors in high-dimensional, correlated datasets. This method has been shown to outperform univariate differential-abundance approaches when the goal is to prioritize predictive features rather than to test for mean differences between groups. Features confirmed as relevant in ≥ 80% of repeated Boruta runs were retained for downstream modeling. To complement the Boruta feature selection, we performed an explainable artificial intelligence analysis using SHAP (SHapley Additive exPlanations) values to quantify the contribution of each taxon to the model’s predictions. SHAP provides model-agnostic, continuous measures of feature importance, allowing interpretation of both the direction and magnitude of each taxon’s effect. Analyses were conducted in R Studio (version 4.3.2 (2023–10-31).) using the iml R package, and summary plots were generated to visualize the relative impact of each taxon on the predicted phenotypes. Multivariable regression models were then constructed to examine the associations between selected taxa and lipoprotein outcomes (LDL particle size, concentrations of lipoprotein subclasses). Taxa abundances were CLR-transformed, and models were adjusted for age, sex, total cholesterol, and adherence to the Mediterranean diet to minimize confounding. Interaction terms (taxon × disease group) were introduced to test for disease-specific associations. Models were implemented in Stata v12 (StataCorp LLC, College Station, TX, USA). Multicollinearity was assessed with variance inflation factors (VIF), and residuals were inspected to confirm model assumptions^80^. A p value of p < 0.05 was considered statistically significant.

## Data Availability

The datasets generated and/or analysed during the current study are available in the NCBI Sequence Read Archive (SRA) repository, under the accession number PRJNA1279632.

## Abbreviations

16S rRNA: 16S ribosomal ribonucleic acid
^1^H-NMR: Proton nuclear magnetic resonance
HDL-C: High-density lipoprotein cholesterol
ApoA1: Apolipoprotein A1
ApoB: Apolipoprotein B
BA: Bile Acids
BMI: Body Mass Index
CD36: Cluster of Differentiation 36
CVD: Cardiovascular Disease
FXR: Farnesoid X Receptor
GGT: Gamma-Glutamyl Transferase
GLP-1: Glucagon-Like Peptide 1
GLP-2: Glucagon-Like Peptide 2
GPT: Glutamate Pyruvate Transaminase
GPR41: G-protein Coupled Receptor 41
GPR43: G-protein Coupled Receptor 43
HbA1c: Glycated Hemoglobin
HDL: High-Density Lipoprotein
HDL-C: High-Density Lipoprotein Cholesterol
IDL: Intermediate-Density Lipoprotein
IDL-C: Intermediate-Density Lipoprotein Cholesterol
IDL-TG: Intermediate-Density Lipoprotein Triglycerides
IL-6: Interleukin 6
LDL: Low-Density Lipoprotein
LDL-C: Low-Density Lipoprotein Cholesterol
LPL: Lipoprotein Lipase
MTP: Microsomal Triglyceride Transfer Protein
MS: Metabolic Syndrome
NMR: Nuclear Magnetic Resonance
PCA: Principal Component Analysis
SCFA: Short-Chain Fatty Acids
SLE: Systemic Lupus Erythematosus
SLEDAI: Systemic Lupus Erythematosus Disease Activity Index
TGR5: Takeda G-Protein Coupled Receptor 5
TG: Triglycerides
VLDL: Very-Low-Density Lipoprotein
VLDL-TG: Very-Low-Density Lipoprotein Triglycerides
